# Detection of Depression and Suicide Risk Based on Text From Clinical Interviews Using Machine Learning: Possibility of a New Objective Diagnostic Marker

**DOI:** 10.3389/fpsyt.2022.801301

**Published:** 2022-05-24

**Authors:** Daun Shin, Kyungdo Kim, Seung-Bo Lee, Changwoo Lee, Ye Seul Bae, Won Ik Cho, Min Ji Kim, C. Hyung Keun Park, Eui Kyu Chie, Nam Soo Kim, Yong Min Ahn

**Affiliations:** ^1^Department of Neuropsychiatry, Seoul National University Hospital, Seoul, South Korea; ^2^Transdisciplinary Department of Medicine & Advanced Technology, Seoul National University Hospital, Seoul, South Korea; ^3^Department of Medical Information, Keimyung University School of Medicine, Daegu, South Korea; ^4^Office of Hospital Information, Seoul National University Hospital, Seoul, South Korea; ^5^Department of Electrical and Computer Engineering and INMC, Seoul National University College of Engineering, Seoul, South Korea; ^6^Department of Psychiatry, Asan Medical Center, Seoul, South Korea; ^7^Department of Radiation Oncology, Seoul National University College of Medicine, Seoul, South Korea; ^8^Department of Psychiatry, Seoul National University College of Medicine, Seoul, South Korea; ^9^Institute of Human Behavioral Medicine, Seoul National University Medical Research Center, Seoul, South Korea

**Keywords:** depression, suicide risk, machine learning, text analysis, objective marker

## Abstract

**Background:**

Depression and suicide are critical social problems worldwide, but tools to objectively diagnose them are lacking. Therefore, this study aimed to diagnose depression through machine learning and determine whether it is possible to identify groups at high risk of suicide through words spoken by the participants in a semi-structured interview.

**Methods:**

A total of 83 healthy and 83 depressed patients were recruited. All participants were recorded during the Mini-International Neuropsychiatric Interview. Through the suicide risk assessment from the interview items, participants with depression were classified into high-suicide-risk (31 participants) and low-suicide-risk (52 participants) groups. The recording was transcribed into text after only the words uttered by the participant were extracted. In addition, all participants were evaluated for depression, anxiety, suicidal ideation, and impulsivity. The chi-square test and student’s *T*-test were used to compare clinical variables, and the Naive Bayes classifier was used for the machine learning text model.

**Results:**

A total of 21,376 words were extracted from all participants and the model for diagnosing patients with depression based on this text confirmed an area under the curve (AUC) of 0.905, a sensitivity of 0.699, and a specificity of 0.964. In the model that distinguished the two groups using statistically significant demographic variables, the AUC was only 0.761. The DeLong test result (*p*-value 0.001) confirmed that the text-based classification was superior to the demographic model. When predicting the high-suicide-risk group, the demographics-based AUC was 0.499, while the text-based one was 0.632. However, the AUC of the ensemble model incorporating demographic variables was 0.800.

**Conclusion:**

The possibility of diagnosing depression using interview text was confirmed; regarding suicide risk, the diagnosis accuracy increased when demographic variables were incorporated. Therefore, participants’ words during an interview show significant potential as an objective and diagnostic marker through machine learning.

## Introduction

Depression and suicide are emerging as important problems worldwide. The lifetime prevalence of depression in the general population has been shown to range between 10 and 15% and has been rapidly increasing over recent decades (1, 2). In addition, unipolar depression is predicted to become the second leading cause of death by 2030 (3). The suicide rate has also increased worldwide by 6.7% over 26 years, and in many European, North American, and Asia-Pacific countries, suicide ranks among the top 10 leading causes of death (4). Moreover, it is known that the risk of death by suicide in people with depression is more than 20 times higher that of the general population, with approximately 15–20% of patients with depression ending their own lives (1, 5).

Early and accurate diagnosis is necessary to implement effective interventions for depression and suicide. However, at present, the only way to diagnose depression and suicidal tendencies is to rely on the patient’s subjective report of symptoms. The diagnosis of depression is made as per the Diagnostic and Statistical Manual of Mental Disorders, Fifth Edition (DSM-V) or International Classification of Diseases and Related Health Problems, 10th Edition (ICD-10). Based on the DSM-V, a major depressive disorder can be diagnosed when five or more depressive symptoms occur and last for two weeks or longer, including (1) depressed mood or (2) loss of interest or pleasure. The ICD-10 diagnostic criteria are similar to those of the DSM and are based on the patients’ subjective report of symptoms (6, 7). Therefore, depression often leads to under-diagnosis in primary care settings, where it is difficult for patients to under-report or have an in-depth interview about symptoms (8, 9). Moreover, in the case of suicide, suicidal behavior disorder was recently defined by the DSM-V but the manual does not specify how to evaluate the actual intention to commit suicide (6, 7). In addition, various scales to assess suicide risk have been developed. Among them, the Columbia-Suicide Severity Rating Scale (C-SSRS) is considered a gold standard for the clinical field (10, 11). However, the gold standard for suicide research remains unclear (12).

By overcoming subjectivity, various attempts have been made to detect and address depression and suicidal ideation. Studies have been conducted to identify depression based on the fact that people with depression exhibit greater hesitation in their voice and a monotonous tone (13). Studies are also underway that aim to predict depression and suicide risk by clustering texts published on social media (14, 15). A study comparing the detection of depression through speech and text showed slightly more accurate results for text (16). Some studies attempt to detect suicide risk early through social media based on the patterns in the texts used by people who committed suicide (17–19). Suicide victims tend to use more the word for a future point in time in their notes than those attempting suicide, and differences in texts were also confirmed, such as expressing positive emotions (20). However, existing studies have mostly been conducted based on written texts, such as social media posts. To the best of our knowledge, no research has been conducted on differences between the texts of patients with depression and those at high risk of suicide based on actual clinical interviews.

Therefore, based on the words used by participants during a semi-structured interview called the Mini-International Neuropsychiatric Interview (MINI), commonly used in the field of mental health, this study established an algorithm that can detect depression and high-suicide-risk groups and examined its accuracy. We hypothesize that there is a difference between the text of people with depression and those with a high suicide risk. The confirmation of our hypothesis can help diagnose depression and predict high-risk suicide cases through artificial intelligence. This study aims to confirm the possibility of using text as an objective marker that can accurately diagnose depression and high suicide risk.

## Materials and Methods

### Participants

Adults aged 19–65 years were recruited among patients with depressive symptoms attending the mood disorder clinic at Seoul National University Hospital from 10 January 2019 to 31 August 2021. A healthy control group was recruited through Internet promotion and notices on nearby campuses. Adults aged 19–65 years, who were healthy and had no depression symptoms, participated in the study. We excluded from our study those: with an impaired ability to independently read and comprehend questionnaires, diagnosed with borderline intellectual disability or dementia, with a history of intracranial surgery, with a history of psychosis, unable to voice themselves due to laryngeal surgery or disease, and cases with significant changes in voice. The participants’ psychiatric diagnosis was confirmed through the MINI. Additionally, in the healthy group, if a previous or present mental illness was found through the MINI, the participant was excluded from the study. Two participants who did not complete the self-report questionnaire were also excluded from this study.

After fully understanding the explanation of the study, all participants signed a consent form to participate in the study, in line with the Declaration of Helsinki. The research procedure was approved by the Institutional Review Committee of Seoul National University Hospital (1812-081-995).

### Assessment

Demographics such as sex, age, height, weight, socioeconomic status (SES), and drugs taken due to non-psychiatric conditions were assessed. Body Mass Index (BMI) was calculated through the recorded height and weight. A precise psychiatric diagnosis was confirmed using the MINI version 7.0.2. Among the items of the MINI, participants with a 1-month suicide-risk assessment and high-degree risk were added to the “depression with high suicidal risk” (DHSR) group. Participants with low, moderate, or no risk of suicide within a month were included in the “depression with low suicidal risk (DLSR)” group.

Antipsychotic drugs taken by the participants can cause changes in speech, such as monotonizing the tone of the voice because of extrapyramidal symptom (21). Therefore, in this study, to consider the effect of antipsychotic drugs, the doses of all antipsychotic drugs being taken were substituted with the olanzapine equivalent dose and summed (22).

Other factors such as depression, impulsivity, and suicidal thoughts were also evaluated. The Patient Health Questionnaire-9 (PHQ-9) was used to assess participants’ subjective depression. The PHQ-9 was developed as a screening scale for depression and comprises nine items rated on a 4-point Likert scale ranging from 0 (*not at all*) to 3 (*nearly every day*). Scores of 10 points or higher indicate moderate to severe depression (23, 24). The Hamilton Depression Rating Scale (HDRS) was used to evaluate objective depression. The HDRS comprises 17 items related to depression severity, and each item was rated using a 5-point Likert scale ranging from 0 (*not present*) to 4 (*severe*). Anxiety was evaluated using the Beck Anxiety Inventory (BAI). The BAI comprises 21 items rated on a 4-point Likert scale ranging from 0 (*not at all*) to 3 (*severely*) (25). Based on the findings of a meta-analysis conducted in 2016, a determination of pathological anxiety was suggested for scores above 16 points (26). When the scores for each item were summed, a score of 17–23 indicated moderate depression, and 24 or higher indicated severe depression (27, 28). Suicide risk was assessed using Beck’s Suicidal Ideation Scale (BSS) (29). The BSS consists of 19 items focused on the intention to commit suicide, and although there is no set cutoff, the higher the score, the higher the suicide risk (17, 30). In addition, since suicide is associated with impulsivity, the latter was assessed using the Barratt Impulsivity Scale (BIS). The BIS-11 consists of 30 questions rated using a 5-point Likert scale ranging from 1 (*never*) to 4 (*always*) (31, 32).

### Text Extraction

During the research, the entire duration of each MINI session was recorded audio; subsequently, only the participant’s words were extracted by tagging the time at which the participant’s speech started and ended. Thereafter, speech shorter than 3 s in length was removed under the assumption that there would be little content, and the remaining speech was segmented into 10-s sections. The segmented speech file was converted into text using a speech recognition toolkit, a Python library. The speech recognizer in the toolkit used Google API, and it was set to “ko-KR” for recognizing Korean speech. For each case, the text of all utterances was recorded independently, and any speech not recognized during this process was dropped. The most widely used Korean preprocessing library, the Konlpy package, was used to extract necessary text information from sentences. Part-of-speech tagging was performed based on a morphological analysis of sentences, and a dataset was constructed by removing stop words (33).

Consequently, the sequence of the words from which the stop word was removed matched to each participant. The model we used in this study used frequency information rather than words’ positional information. Therefore, we again matched all words with each participant after splitting all sentences uttered by the participant into word format. Our model learns the probability of a participant-specific label (depression or risk of suicide) based on the frequency of a word in the data. In the evaluation process, sentences entering the model were separated into words, part-of-speech tagging was performed, stop words were removed, and then matched to the participant again. The model outputs the probability of whether the participant has depression or is at high risk of suicide based on their uttered word sequence.

### Data Analysis

Categorical variables were compared using the chi-square test, and continuous variables were analyzed using student’s *t*-test. Analyses were conducted using IBM SPSS Statistics for Windows (version 25.0; SPSS Inc., Chicago, IL, United States).

The experiment was conducted after building the data pipeline and model pipeline. First, in the data pipeline, fivefold cross-validation was employed. In numerous machine learning studies, K-fold cross-validation is mainly used to verify model rigor and data efficiency. As the interview length differed depending on the participant, the data were divided into the 80% training and 20% test sets based on the participant. First, interviews of the training participants were divided into word units; then, training was carried out according to the label (depression or risk of suicide). At this time, the model was designed based on the Naive Bayes algorithm.

The Naive Bayes classifier is a conditional probability-based machine learning algorithm that calculates the probability of data belonging to each class. In the text domain, the Naive Bayes classifier counts the frequency of words appearing in the entire sentence and then trains a statistical model based on it. In this study, the classifier learned to arrive at a distribution of word frequencies according to the control group and target group (depression and high suicidal risk group). Subsequently, based on the participant’s words, the probability of belonging to each group was predicted.

In particular, for the demographic ensemble model, demographic data, which are structured data, were converted into a probability density function. In this process, because the number of each group is more than 30, a Gaussian normal distribution was assumed (34–37). After generating a Gaussian density distribution from a given demographic bin distribution, the probability of the demographic feature of the target participant belonging to each group was calculated. Subsequently, the ensemble model was built by implementing this probability in the text-based Naive Bayes classifier. The Naive Bayes classifier was used by the Natural Language Toolkit package, and the scikit-learn package was employed for density estimation and analyses (38). All experiments were conducted through fivefold cross-validation.

During training, our model learned word frequency–label relationships. Thereafter, based on what the test participant said in the interview, a prediction based on probability was made. After one training and evaluation in this way, the data were newly split and divided into novel training participant and novel test participant. After this, the model parameters were reset, and training and prediction were performed on the newly split participants. This process was repeated five times, while the data split was performed by setting the participant to be included as a whole in the validation group only once.

## Results

### Comparison Between Healthy Control and Participants With Current Depression

Eighty-three patients with depression were recruited after two patients, who did not submit a self-report questionnaire, were excluded from the analysis. In the healthy control group, 83 participants were evaluated after 22 participants, with a lifetime prevalence of mental illness in the MINI, were excluded from the analysis.

Regarding demographics, the healthy control (HC) group was older and had a lower SES on average, compared to the group with current depression (CD). We also found more people taking other drugs in the CD group compared to the HC group (Table 1). When comparing clinical variables, depression, anxiety, and risk of suicidal ideation were statistically higher in the CD group. However, there was no difference between the two groups in BIS (Table 2).

**TABLE 1:** Demographic comparison of HC and CD groups.

		HC	CD	*P*-value
N		83	83	
Age**	Mean	37.072	30.916	< 0.001
	SD	11.421	10.820	
Sex	F	69 (83.1%)	64 (77.1%)	0.331
	M	14 (16.9%)	19 (22.9%)	
SES*	Low	30 (36.1%)	19 (22.9%)	0.015
	Middle	45 (54.2%)	43 (51.8%)	
	High	8 (9.6%)	21 (25.3%)	
BMI***	Mean	22.364	24.809	< 0.001
	SD	2.902	4.810	
Non-psychiatric medication**	Yes	2 (2.4%)	15 (18.1%)	0.001
	No	81 (97.6%)	68 (81.9%)	

*HC, healthy control; CD, current depression group; N, number; M, male; F, female; SD, standard deviation; SES, socioeconomic status; BMI, body mass index.*

*p < 0.05, **p < 0.01, ***p < 0.001.

**TABLE 2:** Differences in clinical characteristics between HC and CD groups.

		HC	CD	*P*-value
N		83	83	
PHQ***	M	0.892	15.205	< 0.001
	SD	1.440	6.636	
HDRS***	M	4.000	16.638	< 0.001
	SD	3.008	4.805	
BAI***	M	1.542	24.217	< 0.001
	SD	2.923	16.659	
BIS	M	61.470	63.530	0.058
	SD	5.840	7.922	
BSS***	M	1.108	18.145	< 0.001
	SD	1.815	9.531	

HC, healthy control; CD, current depression group; N, number; M, mean; SD, standard deviation; PHQ, Patient Health Questionnaire; HDRS, Hamilton Depression Rating Scale; BAI, Beck Anxiety Inventory; BIS, Barratt Impulsivity Scale; BSS, Beck Scale for Suicidal Ideation. * p < 0.05, ** p < 0.01, *** p < 0.001.

The total number of words spoken by the participants was 21,376, of which rare words, spoken only once, accounted for 4.49%. The maximum sentence length spoken by one participant was 1,504 characters, while the average sentence length was 33.91 characters.

**FIGURE 1:**
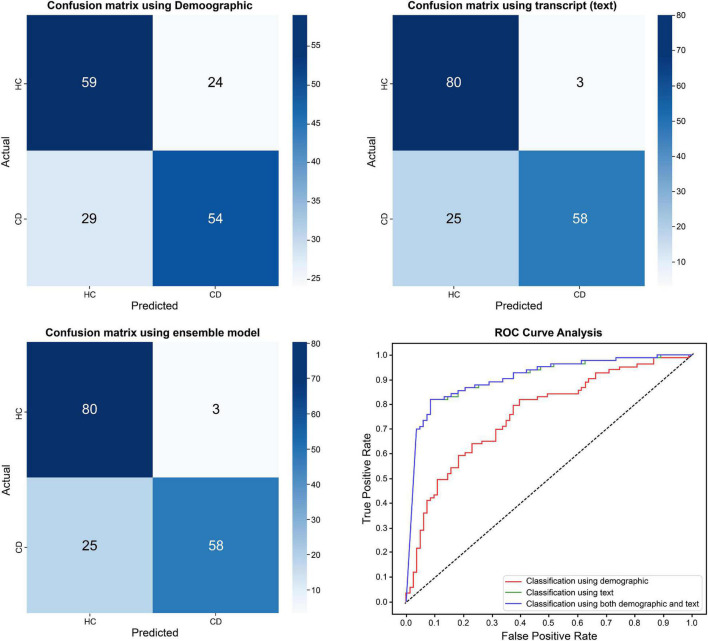
Differences in classification results between healthy control group and current depression group.

**FIGURE 2:**
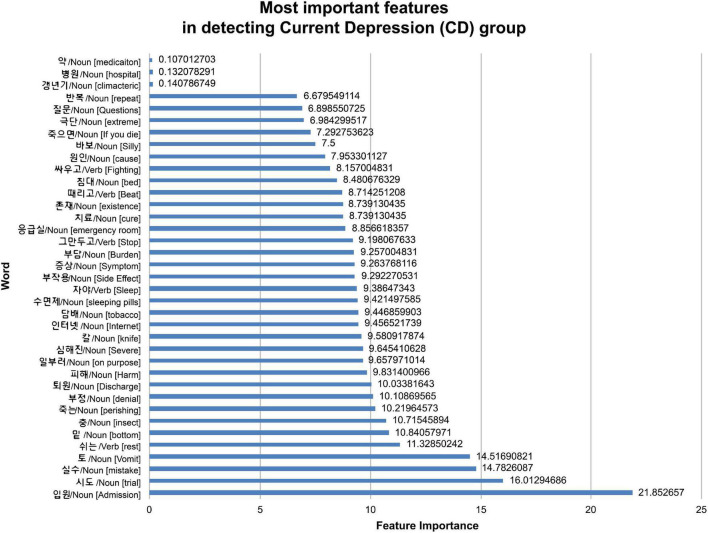
Feature importance for classifying current depression group.

The demographic model for distinguishing between HC and CD included age, SES, BMI, non-psychiatric medication use, and sex, all of which showed statistical differences between the two groups. As for the current depression diagnostic model, the area under the curve (AUC) of the model using demographic information was 0.761, while that of the model using text was 0.902. By contrast, there was no significant difference between the ensemble model trained using demographic and text information and the model trained using only text information (Table 3 and Figure 1). When comparing the ROC curve using demographic data and text through the DeLong test, a *p*-value of 0.001 was confirmed. The statistical significance of this result was confirmed when only demographic data were used and in the ensemble model. By checking the words that the model evaluated as important in the classification process, it was confirmed that the proportion of words with a negative connotation was higher in the CD group (see Figure 2).

**TABLE 3:** Differences in classification results between HC and CD groups.

	Demographic	Text	Ensemble
Accuracy	0.681	0.831	0.831
Sensitivity	0.671	0.699	0.762
Specificity	0.692	0.964	0.951
AUC	0.761	0.905	0.907

*HC, healthy control; CD, current depression group; AUC, area under the curve; Demographic, classification using demographic variables; Text, classification using interview transcript; Ensemble, classification by using both demographics and transcript.*

**FIGURE 3:**
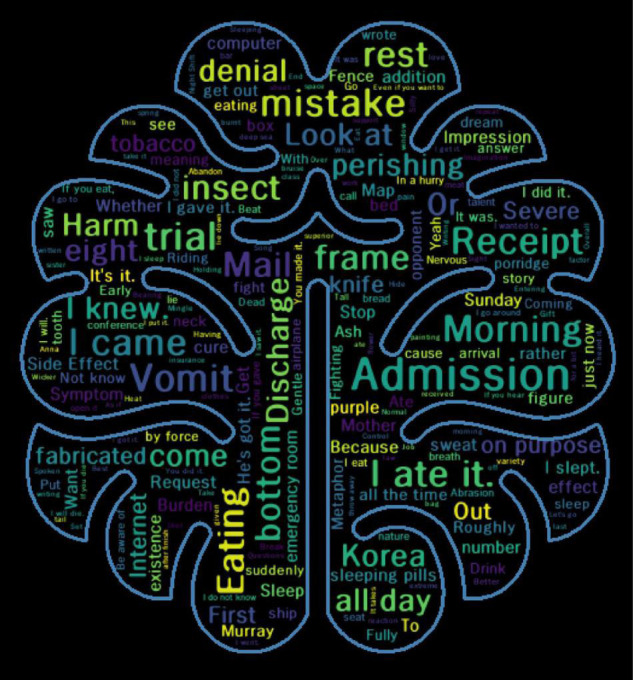
Text mining of important features in current depression group.

In addition, when text mining was performed using words with high frequency, and when the relative importance of the words used by patients with depression was evaluated, the prominent words had a negative connotation, such as “hospitalization,” “mistake,” “negation,” and “floor,” and were found to be widely used (Figure 3).

### Comparison Between Depression With Low Suicide Risk and Depression With High Suicide Risk

Among the 83 patients, 31 were classified as suffering from depression with high suicide risk (DHSR) based on the MINI, and the remaining 52 participants were classified as having depression with low suicide risk (DLSR).

The DHSR group was statistically significantly younger and was on a higher dose of antipsychotic drugs. There were no other differences related to BMI, sex, and SES. When looking at the diagnosis through MINI, the ratios of major depressive disorder and bipolar disorder were similar in both groups, while panic disorder and social anxiety disorder were more common in the DHSR group (Table 4.) As for clinical variables, all indicators of depression, anxiety, and suicidal ideation, except for BIS, were higher in the DHSR group (Table 5).

**TABLE 4:** Differences in demographics between DLSR and DHSR groups.

		DLSR	DHSR	*P*-value
N		52	31	
Age*	Mean	32.865	27.645	0.033
	SD	11.381	9.065	
BMI	Mean	24.366	25.551	0.280
	SD	4.049	5.870	
Sex	M	12 (23.1%)	7 (22.6%)	0.958
	F	40 (76.9%)	24 (77.4%)	
SES	Low	14 (26.9%)	5 (16.1%)	0.209
	Med	28 (53.8%)	15 (48.4%)	
	High	10 (19.2%)	11 (35.5%)	
Non-psychiatric medication	Yes	12 (23.1%)	3 (9.7%)	0.125
	No	40 (76.9%)	28 (90.3%)	
AP_OZP*	N	52	31	0.023
	Mean	4.705	8.082	
	SD	4.872	8.424	
MINI	MDD	7 (13.5%)	4 (12.9%)	0.942
	BP	45 (86.5%)	27 (87.1%)	
	Panic disorder**	2 (3.8%)	8 (25.8%)	0.003
	Social anxiety disorder*	1 (1.9%)	4 (12.9%)	0.040
	OCD	4 (7.7%)	3 (9.7%)	0.753
	PTSD	1 (1.9%)	2 (6.5%)	0.285
	AUD	7 (13.5%)	4 (12.9%)	0.942
	BN	3 (5.8%)	2 (6.5%)	0.899
	GAD	5 (9.6%)	7 (22.6%)	0.104

DLSR, depression with low suicidal risk; DHSR, depression with high suicidal risk; N, number; SD, standard deviation; BMI, body mass index; M, male; F, female; SES, socioeconomic status; AP, antipsychotics; OZP, olanzapine; MINI, Mini International Neuropsychiatric Interview; MDD, major depressive disorder; BP, bipolar disorder; OCD, obsessive-compulsive disorder; PTSD, post-traumatic stress disorder; AUD, alcohol use disorder; AN, anorexia nervosa; BN, bulimia nervosa; GAD, generalized anxiety disorder. *p < 0.05, **p < 0.01, ***p < 0.001.

**TABLE 5:** Differences in clinical characteristics between DLSR and DHSR groups.

		DLSR	DHSR	*P*-value
N		52	31	
PHQ**	Mean	13.462	18.129	0.002
	SD	5.782	7.032	
HDRS**	Mean	15.500	18.548	0.005
	SD	4.734	4.358	
BAI**	Mean	20.577	30.323	0.009
	SD	15.301	17.294	
BIS	Mean	63.981	62.774	0.505
	SD	7.229	9.043	
BSS***	Mean	13.692	25.613	< 0.001
	SD	7.935	7.017	

DLSR, depression with low suicidal risk; DHSR, depression with high suicidal risk; HC, healthy control; CD, current depression group; N, number; M, mean; SD, standard deviation; PHQ, Patient Health Questionnaire; HDRS, Hamilton Depression Rating Scale; BAI, Beck Anxiety Inventory; BIS, Barratt Impulsivity Scale; BSS, Beck Scale for Suicidal Ideation. *p < 0.05, **p < 0.01, ***p < 0.001.

**FIGURE 4:**
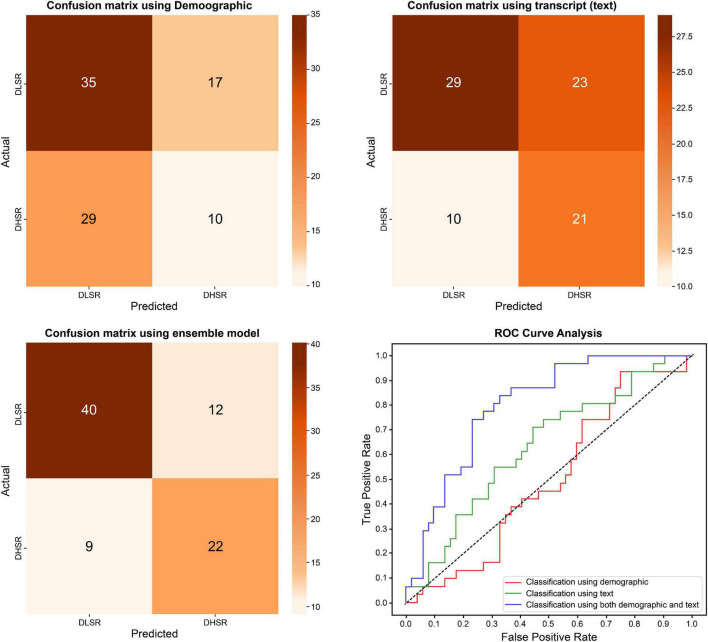
Differences in classification results between depression with low suicidal risk group and depression with high suicidal risk group.

In the model using demographics as a predictor, the DLSR and DHSR groups were classified by including statistically significant differences between the groups related to age, antipsychotic drug dose, SES, BMI, and the BAI score. The AUC of the model trained solely with demographic information was 0.499, showing the lowest accuracy, while the AUC was approximately 0.632 even when DHSR was classified using text, distinguishing between DLSR and DHSR. However, in the case of the ensemble model training, combining the features used in the demographic and text models, the AUC was 0.800, being the highest among all models (Table 6 and Figure 4).

**TABLE 6:** Differences in classification results between DLSR and DHSR groups.

	Demographic	Text	Ensemble
Accuracy	0.542	0.602	0.747
Sensitivity	0.625	0.744	0.816
Specificity	0.370	0.477	0.647
AUC	0.499	0.632	0.800

*HC, healthy control; CD, current depression group; AUC, area under the curve; Demographic, classification using demographic variables; Text, classification using interview transcript; Ensemble; classification using both demographics and transcript.*

## Discussion

This study aimed to diagnose depression based on the words spoken by participants in a semi-structured interview (MINI) and determine whether suicide risk among patients with depression could be predicted based on such textual analysis. We found that the accuracy of detecting depression using speech-converted-text was 83.1%, and the specificity was 96.4%, resulting in better predictions compared to the model using only demographics; the results were statistically significantly different in both groups. In detecting depression, the ensemble model trained with demographics showed similar performance to the model predicting solely through text. However, when predicting the risk of suicide in patients with depression, the sensitivity of the model that predicted high risk solely through text was 74.4%, with its specificity being 47.7%; this result cannot be considered a good performance. In predicting the groups’ suicide risk, when the ensemble model incorporated demographics, better performance was confirmed—a sensitivity of 81.6%, specificity of 64.7%, and AUC of 0.800. This ensemble model performed better than the model based only on demographics. Moreover, the words used in the model to classify depression are clinically noteworthy, such as “hospitalization,” being used by patients themselves. Hence, it is possible to extract important clinically noteworthy features based on machine learning, accumulated by clinicians through experience and assessed based on interviews.

This study is the first to determine whether it is possible to distinguish between healthy groups and those affected by depression, based on the text derived from participants’ speech in semi-structured interviews, and to identify high-suicide-risk groups. Moreover, this study confirmed the diagnosis result by text, developed an algorithm to predict the high-suicide-risk group by integrating text and demographics, and confirmed a surprising AUC value of 79.95%. Previous studies have been conducted based on emotion classification through natural language processing and have mostly performed analysis based on fixed texts written in electronic medical records or social media (39, 40). A previous study predicted depression through a natural language analysis of Twitter posts. The accuracy of predicting depression was 83%, and the F1 score was 0.8329 (41). However, our study showed that the AUC of diagnosing depression using interview text was over 0.9. This is because we employed text taken from an interview with relatively open questions, increasing the possibility of its accuracy. However, when only text was used to predict suicide risk, the AUC was 0.632. This may be due to data imbalance since the number of individuals at suicide risk is only 37.349% among depressed patients. If more data for the patients’ group can be collected, accuracy can be improved.

This study has many strengths, as it is the first to diagnose depression in patients using speech-converted text and to evaluate the risk of suicide. First, the diagnosis was made based on text used in real-time interviews. A toolkit was used to convert the interview into text, making it possible to apply this algorithm in the actual clinical field. In particular, the study can predict depression with high accuracy even without refined data because it uses text that reflects data loss or errors that may occur while transforming the recording file into a toolkit. Second, it builds an ensemble algorithm that can utilize text and demographic data. In this study, using only text data had limitations in predicting groups at high risk for suicide. Therefore, an ensemble model that can utilize various clinical data was constructed. However, to confirm the accuracy of the text, the variables used in the ensemble model were minimized. This ensemble model confirmed its potential as a new diagnostic tool for classifying the risk of suicide. This ensemble model can be proposed as a new objective indicator in psychiatric diagnosis, if elaborated by including more diverse text data and clinical variables. This depression and suicide risk diagnosis algorithm based on artificial intelligence can be seen as a clinical decision support system, and it could help clinicians diagnose depression and suicide risk in various clinical settings, such as when a general practitioner needs to diagnose depression or when visiting the emergency room after an incident related to suicide. An appropriate diagnosis, along with therapeutic intervention, can create more strong therapeutic alliance to better serve patients and caregivers.

There are several limitations to this study. First, the text content used is based on the speech collected from the patients answering the MINI questions, so there is a possibility that critical data were not included. However, it was confirmed that the primary words used by patients with depression were different compared to those used by patients that did not have depression, even if the corresponding question was the same for both groups. This study was conducted based on the text of a semi-structured interview; therefore, further studies based on the speech used in free interviews are required. If data are collected based on a free interview between a clinician and a patient, the range of words used by the patient is likely to be more expansive, and the accuracy of diagnosis through text may decrease. Hence, research is required based on more extensive data, and it is hoped that this study will encourage greater use of the text from interviews for analysis. Second, in the cause-and-effect relationship of the depression discrimination algorithm of this study, whether the text changed due to depression was unclear. Therefore, future studies must confirm the change in text usage patterns according to the change in depression through longitudinal data collection and analysis. Third, as most participants in this study were female (80%) and had experienced bipolar disorder (86.75%), selection bias cannot be ignored. Moreover, since this study was conducted with participants in tertiary medical institutions, words such as “hospitalization” will likely be used. Therefore, it is necessary to confirm whether the results can be replicated based on interview content involving various age groups and clinical sites. Fourth, the amount of text used in this study was small, and deep learning-based analysis could not be applied because the number of participants, especially in the high-risk group, was small. However, meaningful classification was performed only with answers to these limited questions, with the small data size creating a basis for conducting future research with more participants and diverse text content. Fifth, in textual analysis, emotional language classification through natural language processing has been studied extensively. However, such an analysis was not performed in this study due to the limitations in the emotion classification system for Koreans. Although many Korean-based natural language classification datasets have been evaluated, the meaning of Korean words is often reversed depending on the adverb or intonation that follows a word, and many words have not yet been included in the classification. Hence, it could not be used in this study. If the classification of emotional language is more straightforward and can be used for analysis in future studies, the text will be more valuable as a diagnostic tool. Finally, since the text used for analysis did not go through a manual pre-processing evaluation step, there is an error rate to the toolkit, and it may have been evaluated as being lower than the actual rate.

Although limited semi-structured interviews were used, and latest analysis techniques, such as emotional analysis, were not applied, this study confirmed the possibility that the text derived from participant interviews can be an important objective marker for diagnosing depression and detecting suicide risk.

In this study, based on the words spoken by the participants in the MINI interview, depression was detected through machine learning based on the Naive Bayes classifier technique, and the accuracy was confirmed by constructing an ensemble model that predicts the risk of suicide among patients with depression. Detecting depression using text only showed an AUC of 0.905 and predicting high-suicide-risk among such patients showed an AUC of 0.632. In diagnosing depression, speech-converted text showed potential as a good objective marker. In predicting suicide risk, text showed diagnostic utility with an AUC of 0.800 when used with demographics. Whether the results of this study can be replicated will require additional research based on various interviews with more diverse participants.

## Data Availability Statement

The raw data supporting the conclusions of this article are provided upon request from the corresponding author after being reviewed for feasibility.

## Ethics Statement

The study research procedure was approved by the Institutional Review Board of the Seoul National University Hospital (1812-081-995). The patients/participants provided their written informed consent to participate in this study. Written informed consent was obtained from the individual(s) for the publication of any potentially identifiable images or data included in this article.

## Author Contributions

DS, WC, CH, NK, and YA designed the study protocol. DS, MK, and YA recruited the participants. DS, KK, S-BL, CL, and WC analyzed the data and prepared the figures and tables. DS and KK contributed majorly to the manuscript writing. DS, YB, MK, EC, NK, and YA administrated the study. S-BL, YB, MK, CP, EC, NK, and YA edited and revised the manuscript. All authors reviewed the manuscript.

## Conflict of Interest

The authors declare that the research was conducted in the absence of any commercial or financial relationships that could be construed as a potential conflict of interest.

## Publisher’s Note

All claims expressed in this article are solely those of the authors and do not necessarily represent those of their affiliated organizations, or those of the publisher, the editors and the reviewers. Any product that may be evaluated in this article, or claim that may be made by its manufacturer, is not guaranteed or endorsed by the publisher.
